# Lateral ventricle ectopic schwannoma: Case report and literature review

**DOI:** 10.3389/fonc.2023.1090509

**Published:** 2023-01-24

**Authors:** Yujian Li, Xiang Yang, Huiqing Zhou, Jun Zheng, Xuhui Hui, Hao Li, Yanhui Liu

**Affiliations:** ^1^ Department of Neurosurgery, West China Hospital, Sichuan University, Chengdu, China; ^2^ Department of Intensive Care Unit, Fourth People’s Hospital of Sichuan Province, Chengdu, China

**Keywords:** lateral ventricular, ectopic schwannomas, clinical manifestation, pathology, imaging feature

## Abstract

**Background:**

Cases of lateral ventricular ectopic schwannomas (LVES) are extremely rare, with only 23 cases reported thus far. This study aimed to obtain a better understanding of the disease.

**Methods:**

We reported a rare case of LVES, in which the patient was admitted to our hospital, and reviewed the relevant literature on LVES to summarize and analyze the clinical manifestations, pathologies, imaging features and progress.

**Results:**

Of the 23 patients, LVES was more common in men (74%, 17/23) than in women and was mostly located on the right side (78%, 18/23). The average age at clinical presentation was 28 years, with an age range between 8 and 68 years. Moreover, most cases were histologically benign, except in one case of malignancy. In all the benign cases, there were 2 cases of subtotal resection, but no recurrence was found during follow-up.

**Conclusions:**

The origin of LVES could be the tumor transformation of autonomic nerve tissue in the perivascular choroid plexus. For lateral ventricle tumors,which are rare benign lesions with good prognosis after surgical resection, LVES should be considered in the differential diagnosis. Moreover, whether LVES could be considered for gamma knife treatment, similar to a small acoustic neuromas,requires further investigation.

## 1 Introduction

Schwannomas originate from the myelin sheath of peripheral nerves and are mostly benign, accounting for approximately 8% of central nervous system (CNS) tumors. Vestibular schwannoma is the most common, but schwannomas occurring in the brain ventricle or parenchyma are extremely uncommon (1, 2). Ectopic schwannomas (ES) refer to schwannomas occurring in the brain parenchyma or ventricles and are rare in the lateral ventricle (3). The first case of lateral ventricular ES(LVES)was reported by David in 1965 (4), and thus far, only 23 cases have been reported in the English literature. Of these, only one case of malignant biological behavior was reported (5).

Herein, we report a case of right LVES, which was misdiagnosed as a meningioma before surgery. To obtain a better understanding of LVES, this study reviewed the relevant literature on LVES to summarize and analyze the clinical manifestations, pathologies, imaging features and progress.

## 2 Case presentation

### 2.1 Preoperative examination

**Figure 1:**
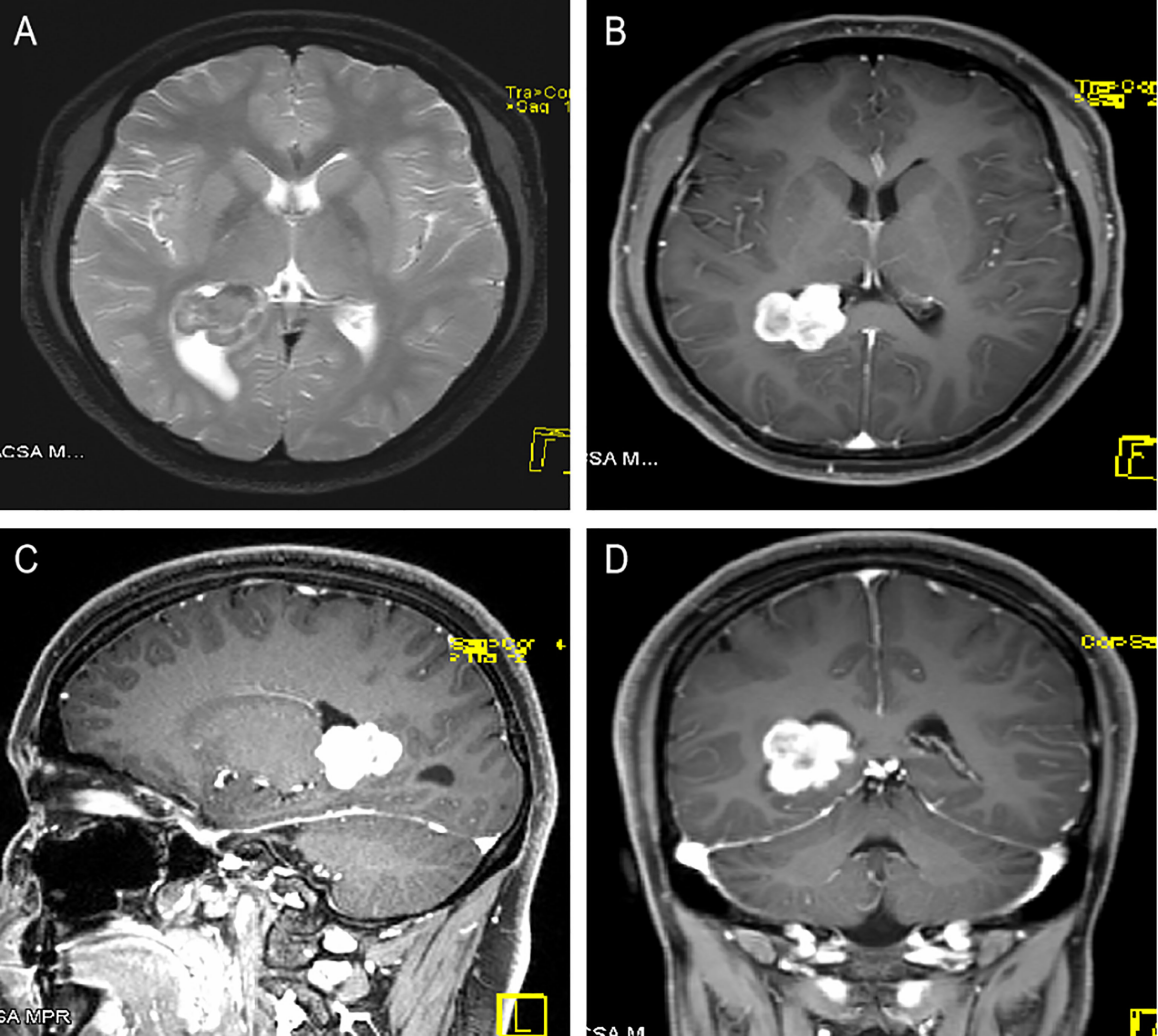
A 22-year-old woman with right LVES. **(A)** Axial T2WI scan showed an approximately 3.0 cm × 2.5 cm × 2.5 cm irregular cystic-solid lesion at the posterior horn of the right lateral ventricle, intimately related to the choroid plexus, with mild peritumoral edema. The solid components exhibited low signa on T2WI, and the cystic components presented hyperintense signals on T2WI. **(B–D)** Axial, sagittal and coronal contrast-enhanced T1WI showed heterogeneous and apparent enhancement of the solid part of the mass but no obvious enhancement in the cystic part.

A 22-year-old Han Chinese woman presented with paroxysmal dizziness, fatigue, nausea and vomiting for 6 years, and the symptoms had worsened over the prior few months. The patient had no previous medical history and no family genetic history of related diseases, and there were no obvious abnormalities on physical examination and laboratory tests, such as blood cell analysis, blood coagulation function, blood biochemistry test, plasmic electrolyte test, cranial nerves examination, motor function and sensory function. Magnetic resonance imaging (MRI) revealed a heterogeneously contrast-enhancing, irregular, lobulated lesion (3.0 cm × 2.5 cm × 2.5 cm in size) at the posterior horn of the right lateral ventricle, and the lesion was intimately related to the choroid plexus. The mass was hypointense on T1-weighted images and iso-hyperintense on T2-weighted images (**Figures 1A–D**). A diagnosis of lateral ventricle meningioma was made before surgery.

### 2.2 Surgical treatment

The patient underwent surgery with the right temporal-occipital craniotomy approach. The intraoperative findings showed that the lesion was irregular and hard and measured 3.0×3.0×2.7 cm in size, and the lesion was closely attached to the choroid plexus. The diagnosis of meningioma was confirmed according to the intraoperative findings.

### 2.3 Postoperative diagnosis and follow-up

**Figure 2:**
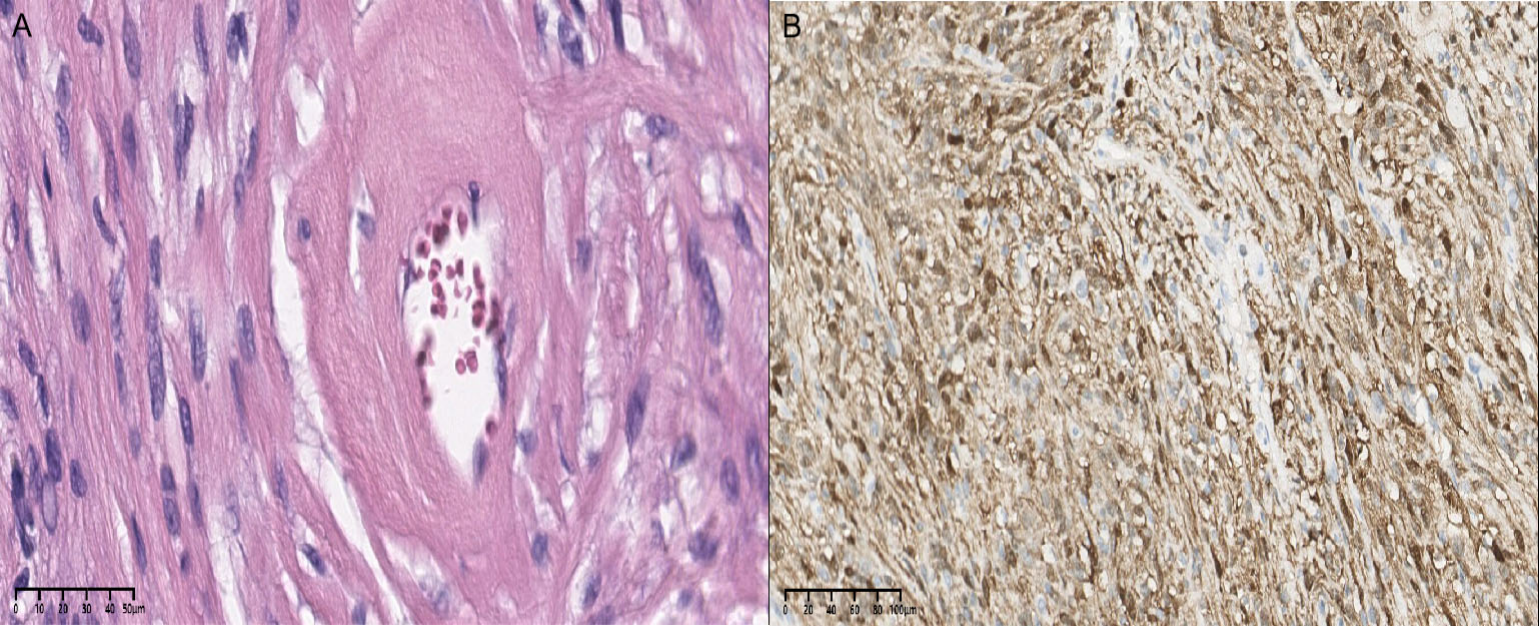
**(A)** Cells are regular, round or spindle shaped, with clear or eosinophilic cytoplasm (H&E×40). **(B)** Diffuse expression of the S-100 protein with immunohistochemistry (×20).

**Figure 3:**
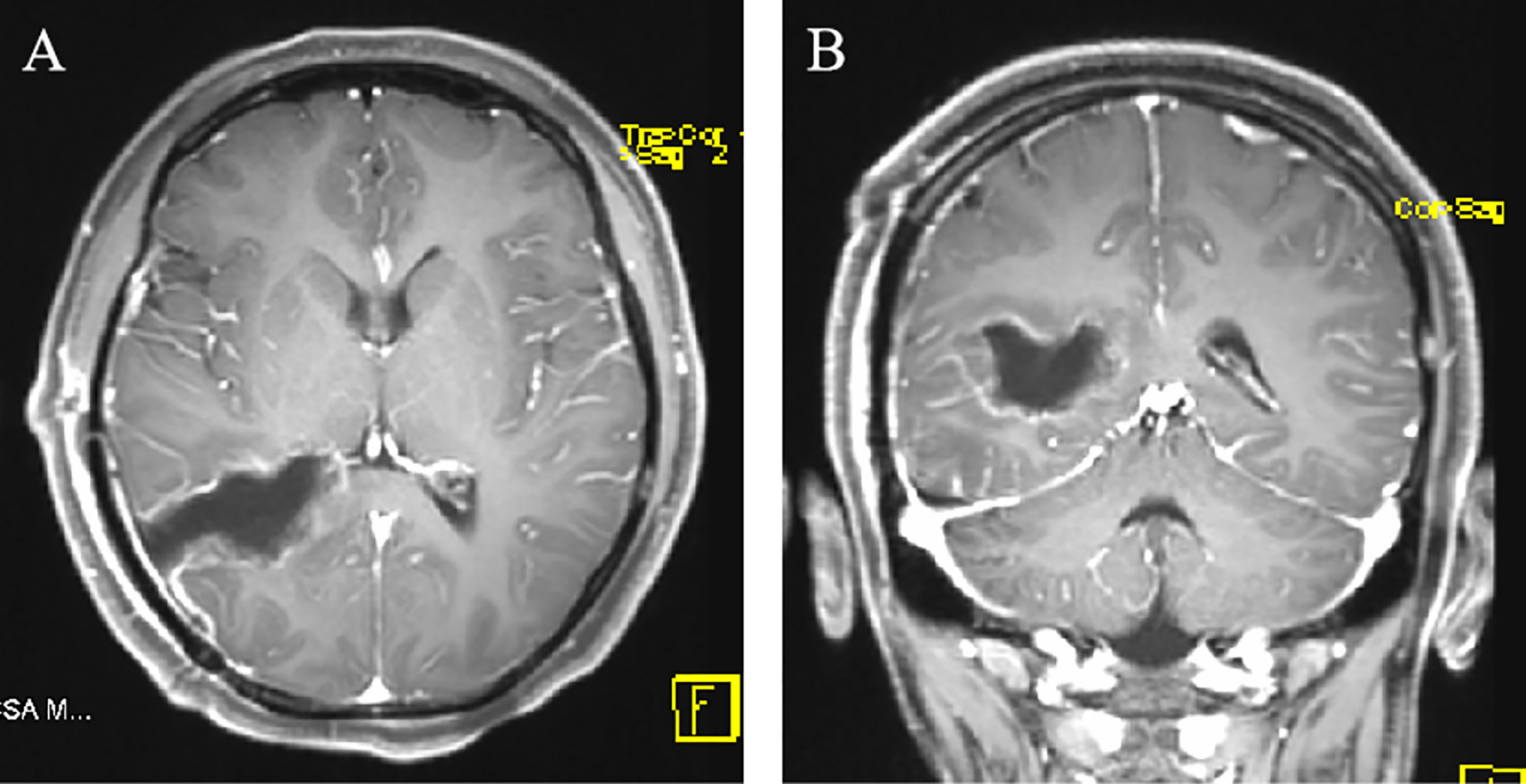
**(A, B)** Postoperative axial and coronal MRI showing that no parts of the right lateral ventricle lesion remained after resection by a temporal-occipital craniotomy.

The lesion was proven to be a schwannoma by pathological analysis (**Figure 2A**). In terms of immunohistochemical staining, the tumor cells were positive for S-100 (**Figure 2B**), vimentin and Ki-67 (1%) and negative for glial fibrillary acidic protein (GFAP) and epithelial membrane antigen (EMA). The postoperative axial and coronal MRI scan revealed that the lesion had been completely resected (**Figures 3A, B**). The patient developed mild depression during the follow-up. The prognosis was goodat the 14-month follow-up.

## 3 Discussion

Schwannomas are benign tumors that arise from nerve sheath cells and are commonly found in the head, neck, and limbs. Intracranial schwannomas account for approximately 8% of CNS tumors. Schwannomas occurring in the brain ventricle or parenchyma are extremely rare (1, 2). LVES is extremely rare, with only 23 cases reported thus far (6).

Although the origin of ES remains unknown, four theories have been proposed: (1) tumor transformation of the autonomic nerve tissue in the perivascular choroid plexus; (2) the transformation of multipotent mesenchymal cells into Schwann cells after tissue injury; (3) the transformation of ectopic fragments of neural crest cells into tumors in the ventricular system during abnormal embryonic development; and (4) the possibility of transformation of mesoderm-derived mesenchymal leptomeningeal cells in the brain into Schwann cells (7–10). In our case, ES was at the posterior horn of the right lateral ventricle, which was closely associated with the choroid plexus during the operation. The tumor transformation of autonomic nerve tissue originating from the perivascular choroid plexus is a reasonable explanation of our case.

**Figure 4:**
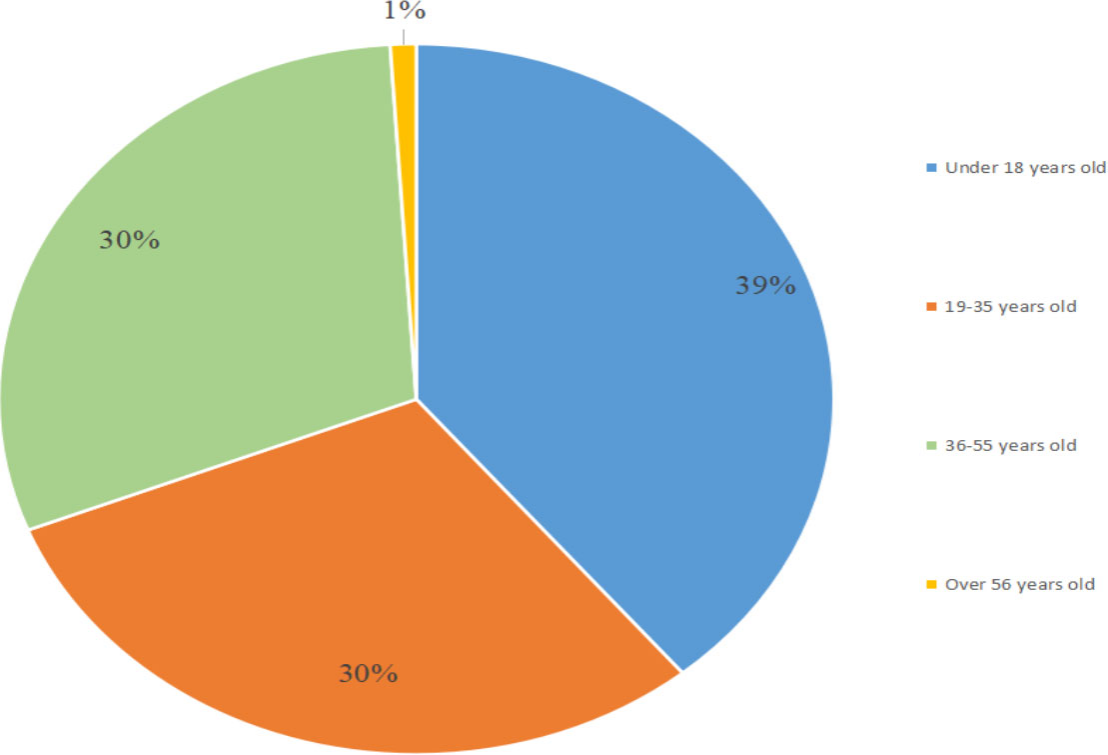
Age distribution of 23 reported cases.

Of the 23 cases (**Table 1**) (4–8, 11–27), LVES was more common in men (74%, 17/23) than in women and was mostly located on the right side (78%, 18/23). The average age at clinical presentation is 28 years, with an age range between 8 and 68 years (**Figure 4**). Moreover, most cases are histologically benign, except in one case of malignancy, in which the patient developed recurrence and metastasis of the tumor (5). The main clinical symptoms of the patient who had malignancy were headache and vomiting, which are similar to the symptoms of most of the reported benign intraventricular schwannomas. Meanwhile, the patient was 40 years old, while we found that 16 of the 22 benign patients were younger than 40 years old. Therefore, malignancy should be suspected in older patients with intraventricular tumors. In addition, the patient was rehospitalized seven months after the first surgery and presented with severe headache and vertigo. Brain MRI revealed tumor recurrence and metastasis. The rapid clinical course was different from the reported cases of benign tumors.

**Table 1:** LVES reported in the English literature.

No.	Year	Author/References	Age/gender	Located in the lateral ventricle (left/right)	Signs and symptoms	Surgical removal result	Benign/malignant	Follow-up
1	1965	David et al. (4)	15/M	R	Headache, vomiting and left hemiparesis	Total	Benign	No Recurrence, 1 year
2	1975	Ghatak et al. (11)	63/F	R	Seizure, facial paresis, hemiparesis	Total	Benign	No Recurrence, 1 year
3	1975	Van Rensburg et al. (12)	21/M	R	Seizure	Total	Benign	No Recurrence
4	1988	Pimentel et al. (13)	8/M	R	Headache, vomiting and left hemiparesis	Total	Benign	No Recurrence, 3 years
5	1990	Ost and Meyer (14)	44/M	L	Right homonymous hemianopsia	Total	Benign	NA
6	1995	Jung et al. (5)	40/M	R	Headache, vomiting, mental status changes	Subtotal	Malignant	Recurrence and metastasis
7	2001	Barbosa et al. (8)	13/F	R	Headache	Subtotal	Benign	No Recurrence, 10 years
8	2003	Erdogan et al. (15)	21/M	R	Left eye visual loss	Total	Benign	No Recurrence, 8 years
9	2004	Dow et al. (16)	16/M	R	Asymptomatic papilledema	Total	Benign	No Recurrence, 1 year
10	2007	Lévêque et al. (17)	16/M	R	Seizure	Total	Benign	No Recurrence, 14 months
11	2008	Benedict et al. (18)	15/M	R	Headache	Total	Benign	No Recurrence, 1 year
12	2009	Vasconcellos et al. (19)	21/F	L	Headache	Total	Benign	No Recurrence, 3 months
13	2013	Luo et al. (20)	24/M	R	NA	Total	Benign	No Recurrence
14	2013	Jaimovich et al. (21)	16/M	R	NA	Subtotal	Benign	No Recurrence
15	2013	Alberione et al. (22)	41/F	R	Headaches and nausea	Total	Benign	No Recurrence, 18 months
16	2015	Glikstein et al. (23)	34/M	L	Seizure, weakness of lower extremity	Total	Benign	No Recurrence
17	2015	Curran-Melendez et al. (9)	20/M	R	NA	Subtotal	Benign	No Recurrence
18	2016	Abdolhosseinpour et al. (7)	9/M	L	NA	Total	Benign	No Recurrence
19	2016	Salazar et al. (24)	16/M	L	Headache and left eye blindness	Total	Benign	No Recurrence
20	2017	Kouitcheu et al. (25)	68/F	R	Headache, vertigo, left hemianopsia	Total	Benign	No Recurrence, 1 year
21	2019	Razak et al. (26)	52/M	R	Subacute headache	Total	Benign	No Recurrence
22	2020	Chiba et al. (27)	26/M	R	Headache and left hemianopsia	Total	Benign	No Recurrence, 6 months
23	2020	Liu et al. (6)	51/F	R	Headache and left hemianopsia	Total	Benign	No Recurrence, 1 year
24	2022	Our case	22/F	R	dizziness	Total	Benign	Good, 14 months

NA, Not Available.

Surgical resection is considered to be curative. Most patients have good results after surgical removal of the tumor (**Table 1**). Of the 21 benign cases reported in previous studies, there was no recurrence of LVES during long-term follow-up after surgery. In our case, the patient, with a completely excised tumor, also had a good outcome after 1 year of follow-up. However, if schwannomas can be accurately identified preoperatively, can gamma knife therapy be considered for small tumors such as small acoustic neuromas?

These tumors cause symptoms that depend on where they are located. The most common symptoms are headache and epilepsy. Of the 23 previously reported cases, except for 4 cases where the symptoms were not recorded, 12 of the patients presented primarily with symptoms such as headache, which may be caused by the mass effect of the tumor (7). In our patient, daily dizziness of gradually increasing intensity, associated with fatigue, nausea and vomiting, was the main clinical manifestation. However, we do not think the patient’s symptoms had much to do with the tumor, or even that the patient’s tumor was an accidental discovery. Unfortunately, the patient was not given more in-depth investigation to support our hypothesis. A review of the clinical features of LVES is shown in **Table 1**.

Immunohistochemical staining is indispensable for the diagnosis of ES. Sometimes it is difficult to distinguish it from meningioma visually and microscopically. S-100 and vimentin are typically positively expressed, while GFAP and EMA are often negatively expressed (7, 25). Through a literature review, we found that some cases appeared as misdiagnoses based on the preoperative and intraoperative frozen section, and the misdiagnoses included ependymoma, cystic astrocytoma, cystic meningioma, hemangioblastoma, fibroblastic meningioma, papilloma and choroid plexus carcinoma (8, 14, 16, 18, 21, 27). In addition, the majority of the 23 cases were diagnosed as LVES based on the pathological findings. Microscopically, the tumor cells can be divided into two types. Antoni A region: cells are often arranged in fusiform; and Antoni B region: cells are often arranged as palisade patterns.

In terms of imaging features, LVES has specific characteristics. MRI is the best diagnostic tool for these tumors because it can be used to determine the location of the ventricles and the relationship between the tumor and choroid plexus.Combined with this case and related literature, these characteristics are summarized as follows. Cystic changes: cystic and solid changes are characteristic of this disease. The cystic part is mostly manifested as low signal intensity on T1WI and high signal intensity on T2WI, while the solid part is often characterized by slightly low signal intensity on T1WI and high signal intensity on T2WI.Moreover, contrast-enhanced MRI showed significant enhancement in the solid but not cystic areas. Edema: peritumoral edema is considered characteristic of benign schwannomas. ES is characterized by peritumoral edema of different degrees (23). Calcification: A previous study reported that part of LVES may exhibit calcification (28), which is helpful for the diagnosis of these tumors to some extent. Among the MRI findings of these 23 LVES cases, cystic changes and edema were more common. Cystic changes were found in 12 patients, and edema was found in 11 patients. Calcification was observed in only 4 patients. In our case, the patient presented with cystic and solid changes and mild edema around the lateral ventricle, and these findings are consistent with previous literature. In addition, whether tumors showing lobulated changes are more likely to be schwannomas is worth considering in future cases.

## 4 Conclusion

The origin of LVES could be the tumor transformation of autonomic nerve tissue in the perivascular choroid plexus. Lateral ventricle tumors are rare benign lesions with good prognosis after surgical resection, and LVES should be considered in the differential diagnosis. Moreover, whether ES could be considered for gamma knife treatment, such as a small acoustic neuroma, requires further investigation.

## Author contributions

All authors contributed to the diagnosis and treatment of the patient. YuL and YX drafted the work and wrote the manuscript. HZ and JZ edited the manuscript, substantively revised it, and approved the re-submitted version. HL and XH provide substantial help to the writing of the article. XH and YuL made substantial contributions to the treatment and diagnosis of the patient. All authors contributed to the article and approved the submitted version.
